# Tumor of the Lower Jaw, the Seat of Vicarious Menstruation, Removal of One-Half of the Bone, Followed by Rapid Recovery

**Published:** 1879-03

**Authors:** Donald Maclean

**Affiliations:** Professor of Surgery, University of Michigan


### TUMOUR OF THE LOWER JAW, THE SEAT OF VICARIOUS MENSTRUATION, REMOVAL OF ONE-HALF OF THE BONE, FOLLOWED BY RAPID RECOVERY.

BY DONALD MACLEAN, M. D., PROFESSOR OF SURGERY, UNIVERSITY OF MICHIGAN.

Miss. E. C. aet. 16, from Assyria, Mich., was admitted to the University Hospital on September 15, 1878, on account of a tumor of the lower jaw, which extended from the symphysis to the ear on the left side. The tumour caused marked deformity of the face and extended into the mouth, pushing the tongue over towards the opposite side. The surface of the growth within the mouth presented the appearance of a fleshy mass, somewhat livid and very vascular. A linear scar from a former operation could be observed at the front and along the lower edge of the tumour. The patient appeared to be in good health in all other respects, but she informed us that she had menstruated through the tumour for two years and had never menstruated in any other way. She stated that the tumour was first noticed six years ago, when it was about the size of a hickory nut, and was situated about the centre of the alveolar margin of the jaw, *i. e.*, midway between the symphysis and the ramus.

She states that for four years the tumour grew very slowly, after which it began to increase rapidly. At that time iodine was applied externally, and, in the patient's opinion, did "a little good." In the spring of 1877 she had a tooth extracted, after which she thinks the tumour diminished in size. Afterwards it began to increase again, and iodine failed to do any good, although freely used.

In September, 1877, Dr. Young, of Nashville, removed the outer plate of the bone on the affected side and scraped away all the diseased structure, but the disease soon began to return and has continued to increase steadily till now. On the 28th of each month the menstrual flow has commenced from the surface of the mass which projects into the mouth, and the quantity has varied from half a pint to a pint each time. The tumour has never been painful.

The length of time that the disease had lasted, the patient's general excellent health, the fact that the tumour was confined to one half of the bone, the absence of pain, all these facts induced me to recommend the operation of excision of the half of the jaw with the tumour and justified me in giving the patient a very hopeful prognosis. (About six years ago I performed a very similar operation here for a young girl of about the same age, and with the most satisfactory results. When heard of, a short time ago, that patient was quite well and presented very slight traces of her operation.)

Chloroform having been administered, I made an incision from a little below the left ear along the base of the tumour to the centre of the lower lip and proceeded to dissect up the flap. At first the hœmorrhage, although not excessive, was very fine, and several quite important branches spouted. Suddenly *all hœmorrhage ceased* and the patient became deadly pale and appeared to be in danger of dying from syncope.

The operation was stopped for a few moments and active efforts were made to revive the patient. Cold water was sprinkled on the face, the feet were raised, and the head lowered, and ammonia was administered by inhalation, and we very soon had the satisfaction of witnessing the disappearance of all the alarming systems. It was thought safer to employ ether during the remainder of the operation, and as the anæsthesia produced by the ether was less profound than that by the chloroform, the patient did not lie as quiet as was desirable. Still no return of the alarming symptoms occurred and no further trouble was met with duiing the operation. The coronoid process was expanded into a broad trunctated cone, so that the division of the temporal muscle was more difficult than usual. Several large arteries required to be ligatured, but the internal maxillary was not wounedd. One harelip suture was used for the lip and the remainder of the wound was closed by interrupted silk sutures. The contour of the face was preserved by filling the gap with lint. There was a good deal of shock, and the patient vomited coagulated blood, which had been swallowed during the operation.

On the evening of the operation the pulse was 160 and very weak, and the temperature $101\frac{1}{2}^{\circ}$

Injections of brandy and hop tea were prescribed and had a good effect. Next day the pulse was 120 and stronger; temperature, 100°. From this time the case made good progress, and on October 9, just one week from the day of operation, she walked into the amphitheatre without assistance. The stitches had all been removed and the wound was almost entirely healed. October 16 she was dismissed cured.—*Michigan Medical News.*

March 9, 1879. The present condition of the subject of the operation above referred to, is worthy of note. Her general health is better than heretofore, with gradual improvement. The parts involved in the operation have entirely healed, with no indication of a return of the tumor, nor is there any discharge from the parts. The natural catamenial discharge has not been established, though every twenty-eight days there is pain characteristic of menstruation in the hypogastric region, with headache. If the uterus, and the associate organs, assume the normal function, doubtless all will be well, if this is not done, time only can determine the result.

This case has several points of especial interest to the dentist, It was an extensive and dangerous operation in oral surgery. It doubtless was much influenced, if not induced, by the teeth, or at least, by a tooth. The mass when removed weighed about one pound; it was somewhat larger, and nearly the shape of the egg of a goose, and some compressed in the direction of its shortest diameter. It was divided through its longest diameter, through the alveolus to the base of the jaw. This separation divided into nearly equal parts, a permanent molar tooth lying at about a right angle with its normal position; the masticating surface looking directly backward, the ends of the roots forward.

This tooth had never erupted, but was simply imbedded in the jaw; and the strong presumption is that it gave rise to this tumor, aided somewhat, doubtless, by the periodical discharges established at this point.

Numerous cases of tumors from impacted teeth are recorded, but I am not aware that any case has been given with all the peculiarities presented in this instance.—Ed. Register.

